# Phase I trial of hypofractionated chemoradiotherapy in the palliative management of esophageal and gastro-esophageal cancer

**DOI:** 10.1186/s13014-022-02127-x

**Published:** 2022-09-14

**Authors:** Swetha Sridharan, Fiona Day, Jasmin Loh, James Lynam, Joanne Smart, Brandan Holt, Hiren Mandaliya, Anthony Bonaventura, Mahesh Kumar, Jarad Martin

**Affiliations:** 1 Department of Radiation Oncology, Calvary Mater Newcastle, Waratah, NSW Australia; 2 Department of Medical Oncology, Calvary Mater Newcastle, Waratah, NSW Australia; 3 School of Medicine and Public Health, University of Newcastle, Callaghan, NSW Australia

**Keywords:** Hypofractionated, Chemoradiotherapy, Esophageal cancer, Dysphagia, Pallative, Efficacy, Clinical trial

## Abstract

**Background:**

Many patients with incurable esophageal cancer (ECa) present with dysphagia as their predominant symptom. Currently there is no consensus on how best to initially manage this scenario with multiple therapeutic options available. We aimed to assess the safety and efficacy of using hypofractionated radiotherapy given over a progressively shorter timeframe with concurrent carboplatin and paclitaxel in the management of patients with ECa and dysphagia.

**Methods:**

In this phase I trial we enrolled patients with histologically proven squamous cell carcinoma or adenocarcinoma of the esophagus or the gastro-esophageal junction with symptomatic dysphagia from local disease and not for curative treatment. Patients needed to be 18 years or older, have an ECOG performance status of 0–2 and be suitable to receive carboplatin and paclitaxel chemotherapy. Patients were placed in four progressively shorter radiation schedules culminating in 30 Gy in 10 fractions in a step wise manner, all with concurrent carboplatin AUC 2 and paclitaxel 50 mg/m^2^ chemotherapy delivered weekly with the radiation therapy. The primary endpoint was the development of the dose limiting toxicities (DLTs) esophageal perforation or febrile neutropenia. Secondary endpoints were relief of dysphagia, time to improvement of dysphagia, dysphagia progression free survival and overall survival.

**Results:**

Eighteen patients were enrolled in the study between October 2014 and March 2019. There were no DLTs experienced during the trial. The most common grade 3 + acute toxicity experienced by patients were nausea and vomiting (both in 4/18 patients). The most common radiation specific acute toxicity experienced was esophagitis with 67% of patients experiencing grade 1–2 symptoms. All patients experienced improvement in dysphagia. The median time to dysphagia improvement was 3 weeks from the start of chemoradiotherapy (CTRT) (range 2–10 weeks). The median dysphagia free survival was 5.8 months with a median overall survival of 8.9 months.

**Conclusion:**

Hypofractionated palliative CTRT with 30 Gy/10# of radiation therapy with concurrent weekly carboplatin and paclitaxel chemotherapy is well tolerated and provides a good response in improvement of dysphagia. Further studies need to be undertaken which provide both symptomatic improvement in the primary tumor but also control of the metastatic burden in these patients.

*Clinical Trial Registration*: This trial was prospectively registered with www.anzctr.org.au Identifier: ACTRN12614000821695.

## Background

Esophageal cancer (ECa) is the 9th most common malignancy globally, however it continues to have a notoriously poor prognosis with 5 year overall survival rate of 15% [1, 2]. For the minority of patients with localized disease suitable for multimodality therapy the outcomes are more favorable [3, 4]. In the United States 35% of patients present with metastatic disease requiring palliative management or best supportive care [5]. Dysphagia is the main symptom of esophageal obstruction and results in significant nutritional deficits, pain and subsequent deterioration in patients quality of life [6].

Dysphagia management is a critical goal of any therapy and allows improved nutritional status and quality of life. This may have a resultant positive impact on overall patient outcomes [7]. There are a number of approaches to dysphagia management including esophageal dilatation, placement of intraluminal stents, systemic chemotherapy, external beam radiotherapy (EBRT), brachytherapy and concurrent chemoradiotherapy (CTRT) [6, 8].

Palliative radiation alone or in combination with chemotherapy is often used to treat dysphagia in patients with incurable ECa. The TROG 03.01 study was a randomized trial of palliative radiotherapy (30–35 Gy/10–15 fractions) with or without concurrent cisplatin/fluorouracil based chemotherapy [9]. It reported a non-significant improvement in dysphagia response in the combined treatment group (45 vs. 35% *p* = 0.13), at the cost of increased grade 3–4 acute toxicity and no difference in overall survival between the two arms. The toxicity of this CTRT regimen alongside the selection of patients with extensive metastatic disease may have contributed to these findings.

In the neoadjuvant setting for patients with curative disease the CROSS study randomized patients to radiation (41.4 Gy/23#) with concurrent carboplatin AUC 2 and paclitaxel 50 mg/m^2^ chemotherapy followed by surgery versus surgery alone [3]. Mature follow-up has shown a median 24 month overall survival advantage for the use of neoadjuvant therapy with manageable toxicity rates [10]. The investigators also reported a complete pathologic response in 29% of the patients who received neo-adjuvant treatment.

Given the tolerability of this CTRT regimen and its favorable outcomes, we conducted a prospective phase I study to assess the safety and efficacy of carboplatin and paclitaxel chemotherapy with hypofractionated radiotherapy in the palliative setting. There is little data regarding the use of hypofractionated CTRT for ECa. The aim was to assess the safety of utilizing this chemotherapy regimen with a progressively more hypofractionated radiation schedule to increase patient convenience in the palliative setting. Here we report the outcomes of this phase I clinical trial conducted at a single institution.

## Methods

### Eligibility criteria

Potentially eligible patients had histologically proven squamous cell carcinoma or adenocarcinoma of the esophagus or the gastro-esophageal junction with symptomatic dysphagia defined by a Mellow score of ≥ 1 (Mellow scale 0 = able to eat all solids, 1 = able to eat only some solids, 2 = able to eat soft foods, 3 = able to drink liquids only, 4 = complete dysphagia) [11]. All patients included were considered to be not amenable to a curative approach with surgery or definitive CTRT due to either patient related factors or advanced disease following discussion in a multidisciplinary forum. Patients needed to be 18 years or older, have an ECOG performance status of 0–2 and assessed suitable to receive carboplatin and paclitaxel chemotherapy with adequate bone marrow function. The inclusion criteria was deliberately kept broad to obtain a signal for response given the Phase I nature of the study. Exclusion criteria included previous thoracic radiotherapy, presence of a tracheo-esophageal fistula, esophageal stent in situ, previous chemotherapy for ECa, presence of bulky or organ threatening metastatic disease thought to require higher dose systemic chemotherapy upfront, and pregnancy. All patients provided written informed consent, and the study was reviewed and approved by the Hunter New England Human Research Ethics Committee (Approval 2019/ETH00815).

### Chemotherapy

Carboplatin was dosed using an area under the curve (AUC) of 2 mg per millilitre per minute and paclitaxel at a dose of 50 mg per square meter body surface area (BSA). Both were administered intravenously on a weekly schedule concurrently in the radiotherapy treatment weeks. Accordingly, in radiation therapy schedules 1, 2 and 3, patients received 3 weeks of chemotherapy. In radiation schedule 4, patients received 2 weeks of chemotherapy.

### Radiation therapy

The biological equivalent dose (BED) was calculated for early reacting tissues using an α/β of 10 and for late reacting tissues using an α/β ratio of 3. The BED for the different radiation schedules were similar to each other and deliberately slightly less than the CROSS regimen to reduce the risk of severe toxicity.

There were four separate radiation therapy schedules:

1. 35 Gy in 15 fractions treating daily over 3 weeks at 2.33 Gy per fraction (BED early α/β = 10 43.1 Gy, BED late α/β = 3 62.1 Gy)
2. 35 Gy in 14 fractions treating over 2.8 weeks at 2.5 Gy per fraction(BED early α/β = 10 43.8 Gy, BED late α/β = 3 64.2 Gy)
3. 33 Gy in 12 fractions treating over 2.4 weeks at 2.75 Gy per fraction (BED early α/β = 10 42.1 Gy, BED late α/β = 3 63.3 Gy)
4. 30 Gy in 10 fractions treating over 2 weeks at 3 Gy per fraction (BED early α/β = 10 39.0 Gy, BED late α/β = 3 60.0 Gy)

As a comparator, the CROSS regimen delivered 41.4 Gy in 23 fractions over 4.6 weeks at 1.8 Gy per fraction (BED early α/β = 10 48.9 Gy, BED late α/β = 3 66.2 Gy).

Patients were accrued to each schedule in a sequential manner starting with schedule 1. Once a minimum of three patients had been accrued to a schedule and there were no dose limiting toxicities (DLT) for each individual patient followed to 6 weeks, the subsequent schedule was opened for recruitment. While awaiting that milestone in the absence of any real time reporting of DLTs, the current schedule remained open to accrual. For schedule 4, once the 3 patients had safely completed the schedule, a further 3 patients were assigned to this regimen to provide greater confidence that this schema was safe and tolerable.

Per protocol, if a DLT was observed in one to two of the three patients in a schedule then a further 3 patients would be required to be enrolled to ensure safely of that schedule. DLTs were defined as per the CTC criteria version 4.03 where patients developed grade 2 or higher esophageal perforation or grade 4 febrile neutropenia.

### Radiation therapy volume and technique

The radiation therapy target volumes comprised of the gross tumour volume (GTV) defined by co-registering a diagnostic FDG PET scan, and internal target volume (ITV) to account for movement of the GTV with respiration, clinical target volume (CTV) which was a further 3 cm superior and inferior margin and 0.5 cm radial expansion. The planning target volume (PTV) was an isotropic expansion of the CTV by 0.7 cm.The dose was delivered to the PTV in accordance with ICRU 50 and 62 with 95% of the isodose to cover the PTV. A 3D conformal radiation technique using 3–4 fields or intensity modulation radiation therapy was permitted to be used.

### Outcomes

The clinical trial primary endpoint was the incidence of dose limiting toxicities, and if this was greater than one third of patients in any schedule, the study would close and the previous schedule would be deemed the maximum tolerated.

Secondary endpoints included:

1. Treatment effect on the relief of dysphagia, with dysphagia relief defined as an improvement in swallowing of at least one point on the 5 point Mellow scale
2. Time to achieving dysphagia relief
3. Dysphagia progression-free survival—Progression of dysphagia was defined as any of the following: a drop of at least 1 point on the dysphagia scale, stricture requiring intervention or death from any cause
4. Changes in patient reported Quality of Life
5. Overall survival times in patients treated with this protocol

### Monitoring and follow up

Pretreatment, all patients were required to undergo a physical examination and assessment of performance status. Baseline blood work (full blood count (FBC), biochemistry including serum urea, creatinine, electrolytes, calcium, and liver function tests) were performed within 2 weeks of study entry. An assessment was made of the baseline dysphagia score using the Mellow scale for all patients as well as documentation of baseline toxicity assessments using the National Cancer Institute (NCI) Common Terminology Criteria for Adverse Events (CTCAE) v.4.03. All patients were also required to have undergone computed tomography (CT) scan of the chest and abdomen, endoscopy and biopsy to confirm diagnosis and staging.

For patients with a baseline Mellow score of 4 (complete obstruction) or those who had lost 20% or more of their normal body weight, parenteral refeeding options such as a Percutaneous Endoscopy Gastrostomy (PEG) tube were considered prior to commencing on study treatment.

During treatment patients underwent weekly physical examination including body weight, performance status, assessment of dysphagia score and toxicity assessment as per CTCAE v.4.03. Full blood counts and biochemistry were checked weekly.

In the follow up period patients were reviewed in clinic at Weeks 5, 9, 13 and 2 monthly thereafter for 12 months. At each visit physical examination, performance status and dysphagia scoring was completed. Other investigations including repeat endoscopy and imaging were undertaken at the physician’s discretion.

### Statistical analysis

The main aim of the study was to explore a novel palliative CTRT regimen for patients with symptomatic oesophageal carcinoma. The primary endpoint was the occurrence of the pre-specified DLTs of grade 2 or higher esophageal perforation or grade 4 febrile neutropenia. Acute toxicity was graded and reported according to the NCI CTCAE v.4.03.

Secondary endpoints focus on efficacy with regard to relief of dysphagia, defined as improvement of at least one point on the Mellow scale. Time to achieving any response in dysphagia was measured from the date of the first radiotherapy fraction. Dysphagia progression-free survival was measured from date of trial enrolment to the time of first progression of dysphagia (at least one point worsening in the Mellow score), stricture requiring intervention or death from any cause. Overall survival is measured from the date of trial enrolment to the date of death, or censored at the date of last follow up. Dysphagia progression free survival and overall survival for the cohort are estimated using the Kaplan–Meier method. For all other data, descriptive results are presented.

## Results

Between October 2014 and March 2019 a total of 18 patients were enrolled in the trial from one institution. Fifteen patients had metastatic disease and three locally advanced tumors, but were not candidates for radical chemoradiotherapy or oesophagectomy. Common sites of metastasis were lymph nodes, liver, lung and bone.

The median age was 68 with a range of 42–81 years. The majority of patients were male with a good performance status of 0–1.The most common histology was adenocarcinoma (13/18) and cancers of the esophagus were more common (13/18) than those of the gastroesophageal junction, all of which were Siewert 1 or 2 (5/18). Seven of the 18 patients had weight loss of greater than 10% of body weight at the time of enrolment. Patient and tumour characteristics are summarized in Table 1.

**Table 1:** Patient and tumour characteristics (n = 18)

Characteristics	Number of patients (%)
Age [years; medium (range)]	68 (42–81)
Gender	
Male	16 (89)
Female	2 (11)
ECOG performance status	
0–1	15 (83)
2	3 (17)
Histology	
Adenocarcinoma	13 (72)
Squamous	5 (28)
Oesophageal tumour site	
Proximal	1 (5.5)
Mid	2 (11)
Distal	10 (55.5)
GOJ	5 (28)
Staging	
Locally advanced	3 (17)
Metastatic	15 (83)
Baseline Mellow score	
1	6 (33)
2	4 (22)
3	5 (28)
4	3 (17)

*ECOG* Eastern Cooperative Oncology Group

Relating to radiotherapy delivered, 14 patients were managed with 3-dimensional conformal radiotherapy (3DCRT), one with intensity modulated radiotherapy (IMRT) and three with volumetric modulated arc therapy (VMAT). The median planning target volume (PTV) was 367 cc (range 240–963 cc) and the median mean combined lung dose was 431 cGy (range 252–1042 cGy).

There were no dose limiting toxicities and we successfully progressed through the planned progressive hypofractionated radiotherapy schedules to schedule 4. An additional patient was enrolled into schedule 1 as one patient did not complete the planned treatment owing to a decline in their condition due to comorbidities. Two extra patients were enrolled into schedule 2 as the last patient had not reached the minimum 6 week follow up but new eligible patients were reviewed and offered trial participation. Treatment schedule details are shown in Table 2.

**Table 2:** Patients in each treatment schedule

Schedule	Number of patients	Chemotherapy cycles
1 35 Gy/15#	4	3
2 35 Gy/14#	5	3
3 33 Gy/12#	3	3
4 30 Gy/10#	6	2

*Gy* gray # - fractions

### Toxicities

There were no dose limiting toxicities. One patient in schedule 1 did not receive the second cycle of chemotherapy due to grade 3 nausea requiring hospitalization following cycle one. The patient subsequently received cycle 3 chemotherapy however suffered a grade 3 hypersensitivity reaction during the paclitaxel infusion. They completed radiation therapy as planned. A second patient on schedule 1 ceased treatment early and did not complete planned chemotherapy and radiation due to grade 3 nausea and vomiting. One patient in schedule 2 died on treatment in the third week. This was not treatment related but due to other co-morbidities including bronchiectasis. The patient developed pneumonia and a spontaneous pneumothorax and failed to respond to treatment measures. Given her incurable disease and rapid decline a decision to palliate was made.

Another patient in schedule 2 died 4 weeks post completion of treatment following general decline in clinical condition. This was deemed due to metastatic disease, and not treatment related.

Five patients in total required hospitalization with nausea and vomiting. All adverse events are shown in Table 3. One of these patients was treated with VMAT, and the remaining four with 3DCRT. The most common side effect was esophagitis with 67% of patients experiencing grade 1–2 symptoms. Only one patient experienced grade 3 acute esophagitis. There were no grade 4 side effects. Grade 1–2 nausea was experienced by 10 of the 18 patients in the trial, with a further 4 patients experiencing grade ≥ 3 nausea. No patient developed severe diarrhea or mucositis. There were no cases of radiation pneumonitis.

**Table 3:** Adverse events of concurrent chemoradiotherapy

Adverse event	Grade 1–2 n (%)	Grade 3 n (%)	Total n (%)
Nausea	10 (56)	4 (22)	14 (78)
Vomiting	3 (17)	4 (22)	7 (39)
Diarrhea	5 (28)	0	5 (28)
Oesophagitis	12 (67)	1 (6)	13 (72)
Mucositis	1 (6)	0	1 (6)
Dermatitis	2 (11)	0	2 (11)
Pneumonitis	0	0	0
Febrile neutropenia	0	0	0
Hypersensitivity	0	1 (6)	0

### Response

Of the 16 patients evaluable at 6 weeks post completion of therapy, 14 (88%) showed an improvement in the Mellow score of at least one point (range 1–4). One patient with complete dysphagia pretreatment reported normal swallowing function at 6 weeks. This patient’s response was sustained and they continued to report normal swallowing function at last review at 50 weeks. Six patients (38%) had an improvement in their Mellow score by 2 points. Of the 6 patients treated in schedule 4 all had an improvement in their Mellow score with 5 of the 6 patients reporting no dysphagia at 6 weeks.

**Fig. 1:**
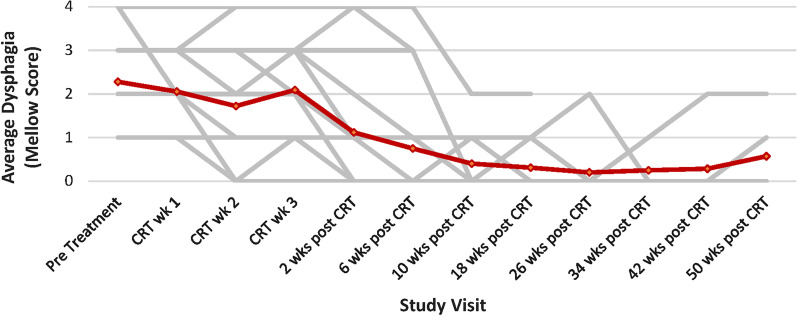
Mellow scores of all patients plotted over time with mean score represented by the curve

**Fig. 2:**
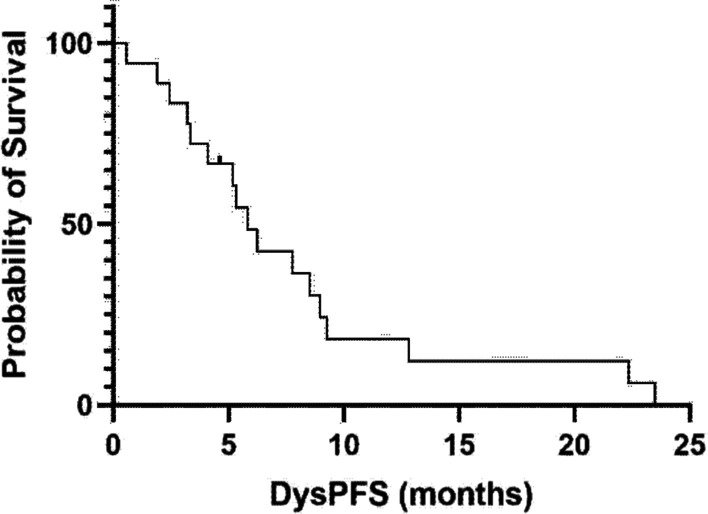
Dysphagia progression free survival

**Fig. 3:**
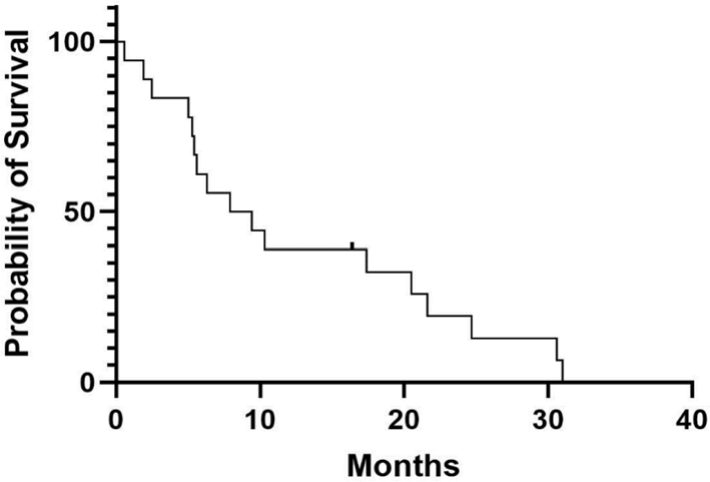
Overall survival

The median time to dysphagia improvement from the start of CTRT was 3 weeks (range 2–10 weeks). Figure 1 shows the individual and mean Mellow scores of patients during and after CTRT, demonstrating improved and durable swallowing improvement for the patient group. The median dysphagia free survival was 5.8 months (Fig. 2) with a median overall survival of 8.9 months (Fig. 3).

### Quality of life (QOL)

Pre-treatment EORTC QLQ–C15 PAL questionnaire was completed by all participants. This QOL tool consists of 15 questions: 2 multi-item functional scales (physical and emotional functioning), 2 multi-item symptom scale (fatigue and pain) and 5 single item symptoms (nausea/vomiting, dyspnoea, insomnia, appetite loss, constipation). There is also a final question on overall QOL. Patients are required to rate each item on the scale from 1 (not at all) to 4 (very much). The global QOL scale is rated from 1 (very poor) to 7 (excellent).

All patients completed baseline questionnaires. The single item symptom of lack of appetite was reported by a third of patients as quite a bit or very poor (6 of 18), with fewer patients reporting nausea at baseline (3 patients reported feeling quite a bit or very nauseated). At baseline 12 of the 18 patients registered a score of 5 or more suggesting a good quality of life.

At 6 weeks post-treatment 16 patients completed the questionnaire. Thirteen of the 16 patients reported the same quality of life score or an improvement of at least 1 point (range 5–7, with median score of 6). Three patients reported a reduction by one point of their quality of life.

Twelve of the 16 patients at 6 weeks reported a stable or improvement in their appetite from baseline. Of the four patients who reported a decline in their appetite only one patient declined to very poor appetite. Similar results were noted for nausea where 13 patients reported the same or better nausea and three reported a worsening of their symptom.

At 18 weeks 11 patients completed the QOL questionnaire as the remainder were deceased. Of the 11, 6 reported a decline in their global quality of life from the score at 6 weeks and the remaining 5 reported stable QOL score.

### Subsequent therapy

Eleven patients proceeded to subsequent 1st line palliative chemotherapy, one of whom had had Cisplatin and Capecitabine prior to study entry. All patients received multiagent chemotherapy with a range of protocols including ECX/ECF, FOLFOX, FLOT, cisplatin/capecitabine, carboplatin/paclitaxel. One patient was found to have a synchronous metastatic non-small cell lung cancer and was started on palliative carboplatin/gemcitabine for this. Another patient received re-irradiation to the esophageal primary tumour for worsening dysphagia 23 months following the first course. He was initially treated on schedule 3 receiving 33 Gy in 12 fractions and received a further 20 Gy in 5 fractions. He presented to hospital with an esophageal perforation 4 months following the subsequent course of radiation and died following developing complications and a middle cerebral artery stroke.

Two patients received stents following local progression after initial treatment, one 15 months and the second 3 months post therapy.

## Discussion

Patients with metastatic ECa have an extremely poor prognosis with the majority of patients succumbing to their disease within 12 months of diagnosis. Given their burden of symptoms and poor prognosis it is critical to manage symptoms effectively including minimizing toxicity and inconvenience of treatment to delay the likely deterioration in quality of life. This phase 1 study confirms the safety and tolerability of using a hypofractionated course of radiation therapy (30 Gy in 10 fractions) with concurrent weekly carboplatin AUC 2 and paclitaxel 50 mg/m^2^ in the palliation of dysphagia.

There were no dose limiting toxicities reported however 22% of patients experienced G3 nausea and vomiting during treatment and one patient experienced G3 esophagitis. These results are slightly lower than the rates of grade 3 + acute toxicity reported in the TROG 03.01 study of 36% in the CTRT arm of the trial. Within the confines of cross trial comparisons, this may be attributable to the chemotherapy, with low-dose carboplatin and paclitaxel being less emetogenic than the 5-FU/cisplatin regimen administered in TROG 03.01.

The radiation volumes in our study followed the protocol used for neo-adjuvant and definitive treatment where the gross tumour volume is expanded to a clinical target volume by 3 cm in the superior and inferior directions. This results in a larger volume of irradiated esophagus and stomach, which may have contributed to the grade 3 nausea and vomiting observed in this phase 1 trial. The management of subclinical disease is less of a priority in the palliative setting, and our current institutional protocol focuses more on the gross disease. We speculate that treatment of the symptomatic primary tumour with a smaller expanded volume should result in lower acute toxicity, particularly less nausea and esophagitis, without compromising the improvement in dysphagia.

The TROG 03.01 study reported a median dysphagia progression free survival of only 4.1 months in the CTRT group [9]. In our small cohort of patients we found a median dysphagia progression free survival of 5.8 months, with the main event being death without a deterioration in dysphagia. This emphasizes that although managing distressing symptoms such as dysphagia is a worthy goal, more effective and tolerable systemic therapy regimens are also critical to optimize outcomes. We also found that all of the 6 patients enrolled into schedule 4 receiving 30 Gy in 10 fractions of radiotherapy with two cycles of chemotherapy had an improvement in their Mellow score, with 5 of 6 patients reporting no dysphagia at 6 weeks. The median time to improvement of dysphagia was 3 weeks for all patients suggesting a good early response.

Management recommendations in this patient group are often based on treatment related factors including tolerability and efficacy, as well as patient and disease related factors including co-morbidities and life expectancy. There is minimal data available which suggests one modality being significantly superior to another when managing dysphagia in patients with advanced disease, with each modality having its own advantages and disadvantages and recommendations being made on a case by case basis. Any palliative treatment in this population should ideally get the balance right between efficacy, convenience and toxicity, prioritizing not placing a significant burden on the patient given their poor prognosis.

Esophageal stenting allows immediate resolution of severe dysphagia with evidence from a large Cochrane meta-analysis of 53 trials and over 3500 patients suggesting self-expandable metal stents achieved better results that other endoscopic procedures [12]. Although a stent can provide immediate relief they may also result in complications including pain, reflux, peri-stent tumour in-growth or stent migration and in some series resulting in up to 50% of patients requiring further endoscopic procedures [13]. In a large retrospective study with nearly 1000 patients, major stent related complications occurred in 20% of patients and 33% were reported to have minor complications [14].

Several randomized controlled trials have compared various treatment approaches in the management of dysphagia. A Dutch study enrolled 209 patients to compare stent placement versus a single fraction of intraluminal esophageal brachytherapy [15]. This study showed a more rapid onset of symptom relief for patients who were stented but a longer period of dysphagia free survival for the brachytherapy patients. This is not a surprising result given the stent has no anti-cancer properties and will therefore eventually experience tumour overgrowth. Other smaller trials of a brachytherapy impregnated stent versus usual stent confirmed similar findings in favour of a longer dysphagia free survival in the brachytherapy patients [16, 17].

A prospective study utilizing brachytherapy alone in 232 patients with locally advanced squamous esophageal carcinomas reported a dysphagia free survival of 7.1 months [18]. In this study 10% of patients developed strictures or fistulas. Brachytherapy for ECa is a specialized technique and hence is difficult to access for many patients. It is also invasive and comes with complications including severe chest pain, fistula formation and risk of aspiration pneumonia. These studies used invasive techniques requiring a high degree of expertise with potentially high rates of severe morbidity is patients with already very poor outcomes. The dysphagia free survival rates are similar to those that we report in this trial with less morbidity.

Apart from the TROG 03.01 study, the majority of the evidence for palliative external beam radiotherapy (EBRT) for esophageal cancer is retrospective with the attendant selection biases. A British retrospective series of patients managed with 50–52.5 Gy in 16–20 fractions of radiotherapy showed the regimen to be tolerable, and achieve similar survival to a conventionally fractionated comparison group [19]. Walterbos et al. retrospectively evaluated three hypofractionated palliative radiation regimens with patients receiving 20 Gy in 5 fractions, 30 Gy in 10 fractions and 39 Gy in 13 fractions. Schedules that used 30 Gy or 39 Gy were associated with a longer duration of response compared to 20 Gy [20]. Response rates with these schedules are as high as 75% however 25–31% of patients eventually required additional treatments for recurrent dysphagia including stent placement or re-irradiation [21]. Welsch et al. reported a retrospective study of 139 patients receiving palliative radiation therapy with external beam doses ranging from 30–40.5 Gy/2.5–3 Gy per fraction, brachytherapy alone or a combination of brachytherapy and EBRT, and found symptom relief in 75% of patients with a median response duration of 5 months [22].

The ROCS trial randomized 199 patients receiving an esophageal stent to the addition of radiotherapy, finding no dysphagia free advantage for the combined group [23]. Overall survival in ROCS was less than 5 months, suggesting that favorable patient selection for multimodal treatment is important. Similarly, radiotherapy alone has long been known to have less efficacy than CTRT in the definitive setting, adding to the hypothesis that for appropriately selected patients, CTRT is a viable treatment strategy [24].

Median overall survival for patients in our small cohort was measured at 7.8 months. Although not a primary outcome of our study, this figure is comparable with published literature and reflects the overall poor prognosis for these patients [9, 22, 25]. Quality of life was another secondary measure we explored. Given the high rate of patient attrition due to disease, and the general expected decline associate with disease progression in this palliative population, it is difficult to make conclusions from this series beyond good early symptomatic benefit being evident.

Given the good tolerability and response achieved from the hypofractionated chemoradiation schedule in this phase I study, a phase II study has been developed. The aim of this Phase II trial (PALEO – PALliative oEsOphageal chemoradioimmunotherapy ACTRN12619001371189) is to manage the symptomatic primary tumour and distant disease in patients with oligometastatic esophageal or gastro-esophageal junction cancer with CTRT and concurrent durvalumab. After completing CTRT to the primary tumour, patients also receive stereotactic radiation (24 Gy in 3 fractions) to a metastasis as an immune primer [26] along with maintenance durvalumab for up to 2 years. The hypothesis is that anti-PDL1 therapy will provide durable control of the metastatic disease, with data emerging to support this in the curative setting and in patients with any burden of metastatic disease treated with platinum-based chemotherapy and anti-PD1 immunotherapy [27, 28].

## Conclusions

The poor outcome for patients with ECa means that a high priority needs to be given to management of patient symptoms without leading to increased burden on them from therapy and its associated toxicity. This study has shown that a 2 week course of radiation along with weekly carboplatin and paclitaxel chemotherapy is well tolerated and provides a good response in improvement of dysphagia. Further studies such as the PALEO trial need to be undertaken to achieve both symptomatic improvement of the primary tumour but also control of the metastatic burden in these patients.

## Data Availability

The datasets used and analyzed during the current study are available from the corresponding author on reasonable request.
